# Multinuclear MRS at 7T Uncovers Exercise Driven Differences in Skeletal Muscle Energy Metabolism Between Young and Seniors

**DOI:** 10.3389/fphys.2020.00644

**Published:** 2020-06-29

**Authors:** Patrik Krumpolec, Radka Klepochová, Ivica Just, Marjeta Tušek Jelenc, Ivan Frollo, Jozef Ukropec, Barbara Ukropcová, Siegfried Trattnig, Martin Krššák, Ladislav Valkovič

**Affiliations:** ^1^High Field MR Center, Department of Biomedical Imaging and Image-guided Therapy, Medical University of Vienna, Vienna, Austria; ^2^Biomedical Research Center, Institute of Experimental Endocrinology, Slovak Academy of Sciences, Bratislava, Slovakia; ^3^Department of Imaging Methods, Institute of Measurements Science, Slovak Academy of Sciences, Bratislava, Slovakia; ^4^Faculty of Medicine, Institute of Pathophysiology, Comenius University in Bratislava, Bratislava, Slovakia; ^5^Christian Doppler Laboratory for Clinical Molecular MR Imaging, Vienna, Austria; ^6^Division of Endocrinology and Metabolism, Department of Internal Medicine III, Medical University of Vienna, Vienna, Austria; ^7^Oxford Centre for Clinical Magnetic Resonance Research, RDM Cardiovascular Medicine, University of Oxford, Oxford, United Kingdom

**Keywords:** magnetic resonance spectroscopy, muscle energy metabolism, saturation transfer, phosphomonoesters, carnosine

## Abstract

**Purpose:** Aging is associated with changes in muscle energy metabolism. Proton (^1^H) and phosphorous (^31^P) magnetic resonance spectroscopy (MRS) has been successfully applied for non-invasive investigation of skeletal muscle metabolism. The aim of this study was to detect differences in adenosine triphosphate (ATP) production in the aging muscle by ^31^P-MRS and to identify potential changes associated with buffer capacity of muscle carnosine by ^1^H-MRS.

**Methods:** Fifteen young and nineteen elderly volunteers were examined. ^1^H and ^31^P-MRS spectra were acquired at high field (7T). The investigation included carnosine quantification using ^1^H-MRS and resting and dynamic ^31^P-MRS, both including saturation transfer measurements of phosphocreatine (PCr), and inorganic phosphate (Pi)-to-ATP metabolic fluxes.

**Results:** Elderly volunteers had higher time constant of PCr recovery (τ_*PCr*_) in comparison to the young volunteers. Exercise was connected with significant decrease in PCr-to-ATP flux in both groups. Moreover, PCr-to-ATP flux was significantly higher in young compared to elderly both at rest and during exercise. Similarly, an increment of Pi-to-ATP flux with exercise was found in both groups but the intergroup difference was only observed during exercise. Elderly had lower muscle carnosine concentration and lower postexercise pH. A strong increase in phosphomonoester (PME) concentration was observed with exercise in elderly, and a faster Pi:PCr kinetics was found in young volunteers compared to elderly during the recovery period.

**Conclusion:** Observations of a massive increment of PME concentration together with high Pi-to-ATP flux during exercise in seniors refer to decreased ability of the muscle to meet the metabolic requirements of exercise and thus a limited ability of seniors to effectively support the exercise load.

## Introduction

With the average age of the earth’s population increasing steadily, incidence of chronic diseases, and prevalence of impairments and disabilities increases as well (Miljkovic et al., 2015). In particular, musculoskeletal disorders, frailty, and sarcopenia leading to a decline in physical functioning are often associated with aging and significantly increase the risk for disability (Brady et al., 2014). The skeletal muscle represents about 40% of the total body mass, and besides its role in maintaining body posture and movement, it also plays a crucial role in regulating whole-body energy metabolism (Frontera and Ochala, 2015). This close link between muscle and whole-body energy metabolism, aging-related impairments in muscle performance, and functional state drives intensive research interests (Choi et al., 2016; Gheller et al., 2016).

While muscle biopsy is the most commonly used diagnostic procedure for myocellular metabolism examination, the invasiveness of this approach generates a strong demand for alternative non-invasive tools. Phosphorous magnetic resonance spectroscopy (^31^P-MRS) has been successfully applied in the past for the non-invasive investigation of skeletal muscle metabolism (Valkovič et al., 2017). In particular, dynamic ^31^P-MRS was previously used to detect prolonged phosphocreatine (PCr) recovery after exercise in aged skeletal muscle (Fleischman et al., 2010; Choi et al., 2016). Muscle phosphodiester (PDE) content also positively correlated with age (Satrustegui et al., 1988; Szendroedi et al., 2011); however, the exact mechanism(s) explaining either of these findings is currently unknown.

^31^P-MRS allows also a direct measurement of metabolic exchange rates and metabolic fluxes, i.e., rate of adenosine triphosphate (ATP) synthesis from inorganic phosphate to adenosine triphosphate (Pi-to-ATP) and ATP resynthesis from creatine kinase (PCr-to-ATP), by the application of saturation transfer (ST) techniques (Valkovič et al., 2017). While these measurements are often performed at rest, the interpretation of resting ST data is very complex and difficult, as it encompasses both oxidative and glycolytic pathways (Brindle and Radda, 1987; Kemp, 2008; Balaban and Koretsky, 2011). Recently, it has been demonstrated that not only dynamic PCr-to-ATP (Goudemant et al., 1997) but also Pi-to-ATP measurements can be performed during an exercise using steady-state-intensity advanced ST techniques, thus providing a new direct measure of the demand-driven ATP synthesis flux (Sleigh et al., 2016; Tušek Jelenc et al., 2016). Further, insights into glycolytic metabolism can be gained through the quantification of phosphomonoester (PME) content at rest and during exercise (Sleigh et al., 2016).

Our primary aim was to test the feasibility of these novel non-invasive methods to detect potential differences in the demand-driven ATP synthesis and resynthesis in the aging skeletal muscle and to determine its relation to PCr recovery and thus ATP production rate following aerobic exercise. The PCr recovery rate is known to be influenced by changes in myocellular pH (Iotti et al., 1993); therefore, our secondary aim was to underpin any potential differences in pH buffering through measurement of muscle carnosine by proton (^1^H) MRS (Just Kukurová et al., 2016). This multinuclear MRS study was performed at 7T to benefit from the increased signal-to-noise ratio (SNR) and improved spectral resolution, provided by the ultra-high field, i.e., 7T (Bogner et al., 2009).

## Materials and Methods

The study population included 15 young (age 29.4 ± 6.7 years; BMI = 21.2 ± 1.8 kg.m^–2^) and 19 elderly volunteers (age 64.6 ± 5.8 year; BMI = 26.7 ± 4.3 kg.m^–2^). Body composition was assessed using bioelectric impedance (Omron-BF511, Japan) between 08:00 a.m. and 09:00 p.m., after an overnight fast. All subjects had no record of musculoskeletal or cardiovascular disease and were asked to restrain any physically strenuous activity one day prior to MRS examination. Written informed consent was signed by each participant prior to entering the study, and the study was performed according to the Declaration of Helsinki with an approval of the appropriate ethics committees.

All measurements were performed on a 7T whole-body MR system (Siemens Healthineers, Erlangen, Germany). Participants were positioned supine with their right calf placed in a 28-channel knee coil (QED, Mayfield Village, OH, United States) for ^1^H-MRS acquisition first. Afterward, participants were repositioned onto an MR-compatible ergometer (Trispect, Ergospect, Innsbruck, Austria) with the ^31^P/^1^H surface coil (10 cm, Rapid Biomedical, Rimpar, Germany) positioned under the right calf for ^31^P-MRS. Both measurement protocols are described in more details below.

### ^1^H MRS

**FIGURE 1:**
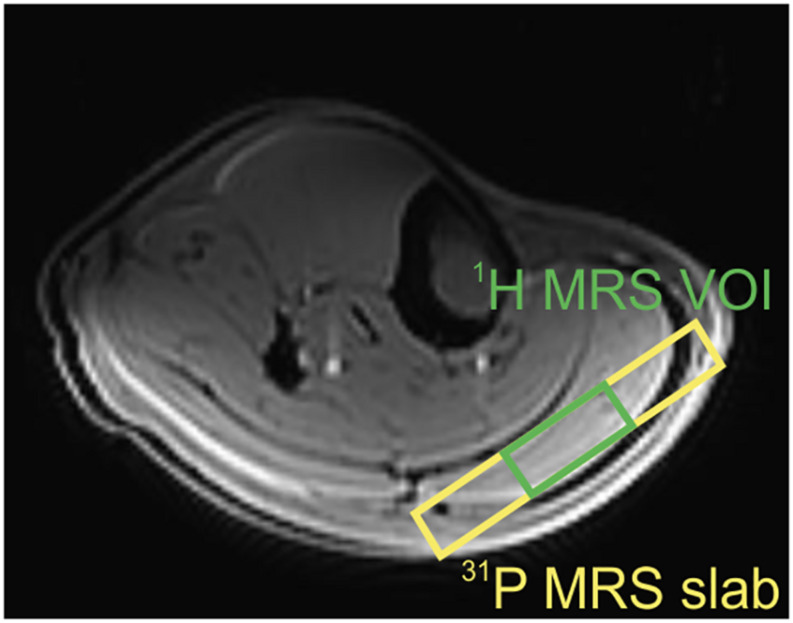
Transversal slice of the T_1_-weighted localizer image with representative volume-of-interest (VOI) positions in the gastrocnemius muscle. Green VOI depicts the ^1^H MRS volume, and yellow VOI reflects the slab for ^31^P MRS acquisition.

Based on T_1_-weighted, multi-slice localizer images, the volume of interest (VOI) of 40 × 30 × 12 mm^3^ was selected in the gastrocnemius muscle using a STEAM localization sequence (Figure 1). Localized shimming was performed manually, on the adjustment volume that matched the VOI, after automatic field map acquisition based on gradient recalled, and double-echo field map acquisition (GRE-SHIM, Siemens Healthineers). The final linewidth of the water signal was in the range of 28–38 Hz in the magnitude mode. The MR signal of carnosine was measured with 80 Hz water suppression and the following parameters: TR = 9000 ms, TE = 20 ms, spectral bandwidth = 3 kHz, NA = 64, delta frequency = 2.8 ppm, i.e., centered on the carnosine signal resonating at 8 ppm. The water signal, which was used as a concentration reference, was measured in a separate non-water suppressed acquisition from the same VOI using following parameters: TR = 2000 ms, TE = 20 ms, NA = 1, and delta frequency = 0 ppm.

### ^31^P MRS

Depth-resolved *in vivo* spectroscopy (DRESS) was used for signal localization in all static and dynamic ^31^P MRS experiments (Valkovič et al., 2014). A 15-mm-thick selection slab representing the VOI was placed over the gastrocnemius muscle (Figure 1), and the shimming was performed manually. The RF transmit voltage was adjusted based on calibration measurements with increasing RF power and searching for the maximum of the localized PCr signal.

First, a long TR ^31^P MR spectra (TR = 15 s, NA = 8, and FA = 90°) were acquired during 2 min at rest. Four-angle saturation transfer (FAST) measurements taking 3.5 min were performed as previously described (Tušek Jelenc et al., 2016). Briefly, the first experiment applied a nominal FA of 52° and NA = 8, and the second experiment applied a nominal FA of 15° and NA = 24. Saturation was applied first at the γ-ATP frequency, and the control saturation mirrored around PCr for PCr-to-ATP and around Pi for Pi-to-ATP reactions.

Next, the dynamic experiment was performed as described previously (Tušek Jelenc et al., 2016). The dynamic measurement consisted of 2 min of rest for baseline data, 6 min of plantar flexion exercise, and 6 min of recovery phase. The exercise was performed at a workload of approximately 30% of maximal voluntary contraction (MVC, assessed prior to MRS examination by a measurement of contraction force during repeated isometric pushing against a blocked ergometer pedal) force with one flexion per TR (TR = 2 s). FAST measurement was repeated during exercise starting 2 min after exercise onset, when the steady state of PCr depletion is typically reached.

### Data Analysis

All acquired spectra were fitted using time domain fitting routine AMARES in jMRUI (Vanhamme et al., 1997). The resonance lines of PCr, Pi, and Pi_2_ were fitted as single Lorentzians; α- and γ-ATP signals were fitted as doublets and β-ATP as a triplet. Due to the non-conventional line shapes of the PME and PDE resonances, concentrations of both (PME and PDE) were determined by integration techniques. The concentration of PMEs included concentrations of phosphoethanolamine (PE), phosphocholine (PC), hexose monophosphates, e.g., glucose-6-phosphate (G6P), fructose-6-phosphate (F6P), and inosine monophosphate (IMP), whereas the concentration of PDEs was based on concentrations of glycerol-phosphocholine (GPC), and glycerol-phosphoethanolamine (GPE). The Pi_2_ peak frequency was constrained with respect to its expected shift to Pi to ∼0.4 ppm. Intramyocellular pH was calculated according to the modified Henderson–Hasselbalch equation, based on the chemical shift of PCr and Pi signals. The γ-ATP peak was used as an internal concentration reference, assuming a stable cellular ATP concentration of 8.2 mM (Taylor et al., 1983).

For the FAST experiment, the fully relaxed magnetizations of PCr and Pi (M_0_), their partially saturated magnetization (M_0_’), and the apparent longitudinal relaxation time (T_1_^*app*^) were calculated according to Bottomley et al. (2002). The pseudo-first-order exchange rate constants of the Pi-to-ATP (k_*ATP*_) and PCr-to-ATP (k_*CK*_) reactions were computed as k = 1/T_1_^*app*^ × (1–M’_0_/M_0_), and the forward exchange fluxes were calculated as *F*_*ATP*_ = *k*_*ATP*_×[*Pi*] (or *F*_*CK*_ = *k*_*CK*_×[*PCr*]).

The time constant of PCr resynthesis (τ_*PCr*_) was calculated based on the monoexponential fitting of the PCr recovery curve. The initial rate of PCr resynthesis (V_*iPCr*_) during the recovery period was used to calculate the maximal rate of oxidative phosphorylation (Q_*max*_) according to the adenosine diphosphate (ADP)-based model of Michaelis and Menten $Q_{\max}=V_{i\,P\,C\,r}+\left(\frac{K_{m}}{{\left[A\,D\,P\right]}_{e\,n\,d\,\_\,e\,x\,e\,r\,c\,i\,s\,e}}\right)$. The ADP concentration was calculated according to the method described by Kemp et al. (1993), assuming that 15% of total creatine was not phosphorylated in the resting state.

The C_2_-H peak of carnosine, resonating at 8 ppm, was fitted after removing residual peaks of water and lipids by a Hankel Lanczos squares singular values decomposition (HLSVD). Using the water signal as an internal reference, the concentration of carnosine was calculated according to the formula for millimolar concentrations in 1 kg of wet weight of tissue (mmol/kg ww): $c_{c\,a\,r}=c_{H_{2}\,O}\times \left(\frac{S_{c\,a\,r}}{S_{H_{2}\,O}}\right)\times \left(\frac{n_{H_{2}\,O}}{n_{c\,a\,r}}\right)\times \left(\frac{C\,F_{H_{2}\,O}}{C\,F_{c\,a\,r}}\right)\times w_{H_{2}\,O}$, where *S* is the signal intensities, n_*H*__2__*O*_ is the number of corresponding equivalent protons in water (*n* = 2), CF is the correction factor for *T*_1_ and *T*_2_ relaxation times (Just Kukurová et al., 2016), c_*H*__2__*O*_ = 55.56 mol/l is the concentration of the water, and w_*H*__2__*O*_ is the approximate water content of skeletal muscle, i.e., 0.77 l/kg wet weight of tissue.

Data are presented as mean ± standard deviation. Comparisons between parameters acquired at rest and during exercise within subject groups were performed using a paired Student’s *t*-test, and the comparison of the two subject groups was performed using an unpaired Student’s *t*-test with a Benjamini–Hochberg adjustment for multiple comparisons (α = 0.1). The relations between parameters were analyzed by linear correlation using Pearson’s correlation coefficient to estimate the strength of the relationships. The results were considered as statistically significant at *p* < 0.05 (*t*-test).

## Results

**FIGURE 2:**
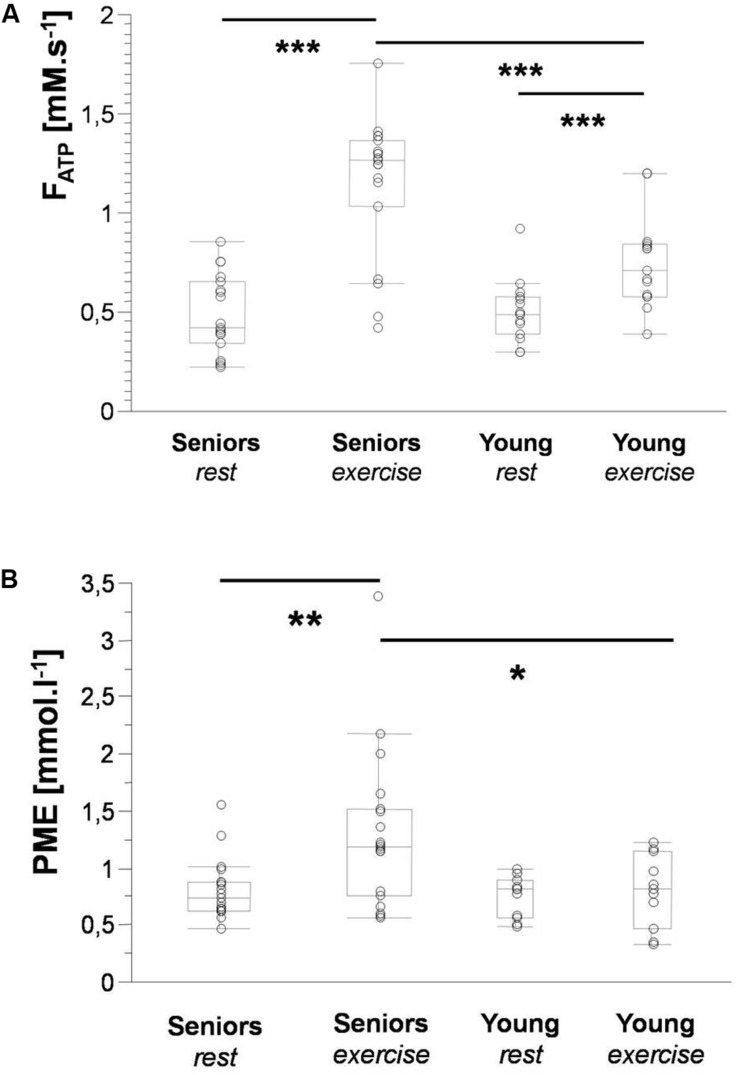
Box plots depicting significant differences in Pi-to-ATP flux **(A)** and PME concentration **(B)** between seniors and young volunteers at rest and during exercise. Data are presented as the median with the lower and upper quartiles; whiskers represent minimal and maximal values. Significance: * < 0.05; ** < 0.01; and *** < 0.001.

**FIGURE 3:**
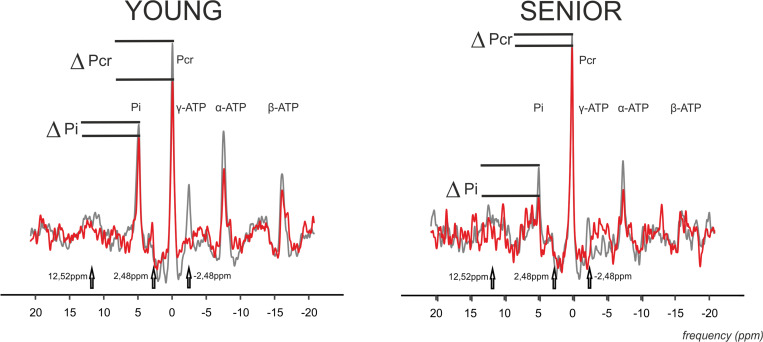
Representative ^31^P MR spectra acquired using four-angle saturation transfer (FAST) experiment with FA = 52° during exercise in the gastrocnemius medialis muscle of young and senior participants. Spectra with γ-ATP saturation (-2.48 ppm) are drawn as a red line, and the controlled spectra are drawn as a gray line. The controlled spectra are a combination of two control saturations connected at 2 ppm, i.e., the control experiment for the PCr-to-ATP exchange rate at a downfield frequency mirrored around PCr at 2.48 ppm, and with the control experiment for the Pi-to-ATP exchange rate at a downfield frequency mirrored around the Pi resonance at 12.52 ppm. Please note the drop in PCr and Pi signal caused by the chemical exchange.

The time constant of PCr recovery (τ_*PCr*_) was significantly higher in the senior group (43.69 ± 11.04 s) in comparison to the young group (29.29 ± 8.26 s; *p* = 0.0008). A significant decrease in PCr-to-ATP flux was observed during exercise from 5.92 ± 1.15 mM.s^–1^ to 2.42 ± 1.15 mM.s^–1^ (*p* = 0.0001) in the elderly group and from 6.87 ± 1.38 mM.s^–1^ to 3.79 ± 0.93 mM.s^–1^ (*p* = 0.0001) in the young group. The difference between the two groups was significant at rest as well as during exercise. Similarly, the increase in Pi-to-ATP flux (ΔF_*ATP*_) during exercise was also significant (0.49 ± 0.20 mM.s^–1^ vs. 1.15 ± 0.35 mM.s^–1^, and *p* = 0.0001, in seniors and from 0.51 ± 0.15 mM.s^–1^ to 0.76 ± 0.23 mM.s^–1^, and *p* = 0.002, in the young group; Figure 2A). Elderly subjects differed significantly (*p* = 0.0006) from young subjects in muscle F_*ATP*_ flux only during exercise, not at rest (*p* = 0.77). Representative spectra acquired during exercise using the FAST experiment are shown in Figure 3.

**FIGURE 4:**
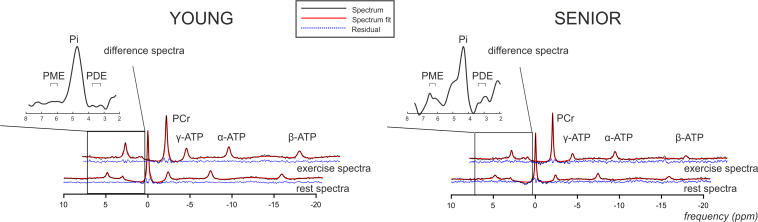
Representative rest and exercise ^31^P-MR spectral examples acquired from the gastrocnemius medialis muscle (black lines) from young and senior participants showing final spectral fit (red lines) and the residual (blue dashed lines). Please also note the magnified difference spectra of the region around Pi.

A higher concentration of PME during exercise was observed in the muscle of the elderly subjects than in the young subjects (Figure 2B). Moreover, significant was also the increase in PME associated with exercise (ΔPME) in the elderly group (*p* = 0.006) but not in the young group (*p* = 0.62; Figure 4). The difference in muscle PDE content between young and seniors was significant both at rest and during exercise, but the exercise-related decrease was not found significant in either group.

**FIGURE 5:**
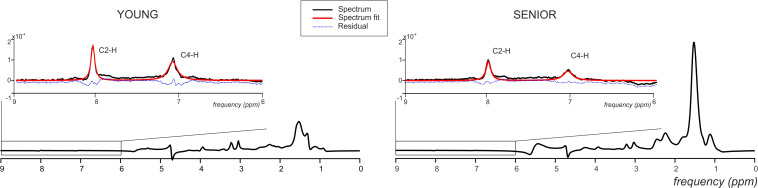
Representative ^1^H MR spectra acquired from the gastrocnemius medialis muscle from young and senior participants with a magnified carnosine area showing spectral examples (black lines), final spectral fit (red lines), and the residual (blue dashed lines).

Seniors differed significantly from young volunteers also in concentration of carnosine (*p* = 0.0004) when its concentration in muscle of seniors achieved only 58.09% of that found in muscle of young volunteers. Detailed results are summarized in Table 1. Exemplary spectra are given in Figure 5.

**TABLE 1:** Characteristics of the study population and results of ^1^H and ^31^P MRS.

	Seniors		Young		*t*-test *p*-value	B-H step-up (α = 0.1)
	Mean ± SD	N	Mean ± SD	N		
**Anthropometry**						
Age (y)	64.58 ± 5.84	19	29.4 ± 6.72	15	**<0.0001**	**0.003**
Weight	72.53 ± 12.83	19	64.68 ± 9.31	11	0.0829	0.071
BMI (kg.m^–2^)	26.65 ± 4.30	19	21.15 ± 1.79	11	**0.0004**	**0.014**
Fat mass (%)	35.48 ± 10.3	19	25.52 ± 8.53	9	**0.0184**	**0.054**
Muscle mass (%)	27.13 ± 5.12	19	31.52 ± 8.10	9	0.0913	0.074
**Metabolic concentrations and fluxes at rest**						
Carnosine (mmol.l^–1^)	3.75 ± 1.56	19	6.43 ± 1.76	9	**0.0004**	**0.017**
PME (mmol.l^–1^)	0.82 ± 0.27	19	0.76 ± 0.18	11	0.5273	0.094
Pi (mmol.l^–1^)	4.26 ± 0.94	19	4.03 ± 0.59	15	0.402	0.091
GPC (mmol.l^–1^)	5.35 ± 1.63	19	2.96 ± 0.64	11	**<0.0001**	**0.006**
PDE (mmol.l^–1^)	5.92 ± 1.46	19	3.94 ± 0.61	11	**0.0002**	**0.011**
PCr (mmol.l^–1^)	30.65 ± 0.81	19	30.19 ± 0.83	11	0.1472	0.077
pH	7.08 ± 0.03	19	7.11 ± 0.02	11	**0.0101**	**0.043**
NADH (mmol.l^–1^)	0.81 ± 0.55	18	0.58 ± 0.29	11	**0.014**	**0.046**
kATP (s^–1^)	0.10 ± 0.04	19	0.13 ± 0.03	15	**0.0203**	**0.057**
FATP (mM.s^–1^)	0.49 ± 0.20	19	0.51 ± 0.15	15	0.7719	0.100
kCK (s^–1^)	0.20 ± 0.04	19	0.23 ± 0.04	15	**0.0252**	**0.060**
FCK (mM.s^–1^)	5.92 ± 1.15	19	6.87 ± 1.38	15	**0.0361**	**0.066**
**Metabolic concentrations and fluxes during exercise**						
PME (mmol.l^–1^)	1.31 ± 0.69	19	0.81 ± 0.32	11	**0.0338**	**0.063**
PDE (mmol.l^–1^)	5.43 ± 1.82	19	3.56 ± 1.13	11	**0.0049**	**0.040**
Pi (mmol.l^–1^)	15.88 ± 5.94	19	13.10 ± 5.53	15	0.1725	0.083
PCr (mmol.l^–1^)	16.01 ± 6.63	19	23.1 ± 4.72	15	**0.0014**	**0.034**
PCr drop (%)	47.9 ± 21.9	19	23.08 ± 14.85	15	**0.0007**	**0.026**
pH	7.03 ± 0.06	19	7.08 ± 0.01	11	**0.0183**	**0.049**
kATP (s^–1^)	0.08 ± 0.03	19	0.06 ± 0.03	15	0.1494	0.080
FATP (mM.s^–1^)3	1.15 ± 0.35	19	0.76 ± 0.23	15	**0.0006**	**0.020**
kCK (s^–1^)	0.15 ± 0.04	19	0.17 ± 0.04	15	0.2278	0.086
FCK (mM.s^–1^)	2.42 ± 1.15	19	3.79 ± 0.93	15	**0.0007**	**0.029**
**Recovery-derived parameters**						
tau (s)	43.69 ± 11.04	19	29.29 ± 8.26	11	**0.0008**	**0.031**
Qmax (mM.s^–1^)	0.49 ± 0.15	19	0.45 ± 0.10	15	0.3276	0.089
VPCr (mM.s^–1^)	0.35 ± 0.15	19	0.19 ± 0.05	15	**0.0025**	**0.037**
PCr (mmol.l^–1^)	30.88 ± 0.94	19	30.74 ± 1.24	11	0.7263	0.097
Pi (mmol.l^–1^)	4.28 ± 0.98	19	3.56 ± 0.69	11	**0.0415**	**0.069**
pH	7.02 ± 0.03	18	7.09 ± 0.05	11	**<0.0001**	**0.009**
PME (mmol.l^–1^)	0.94 ± 0.42	19	0.61 ± 0.16	11	**0.0183**	**0.051**
PDE (mmol.l^–1^)	5.9 ± 1.7	19	3.81 ± 0.68	11	**0.0006**	**0.023**

*Data are presented as mean ± standard deviation. Unpaired Student *t*-test was performed for comparison between young and senior participants. *p* < 0.05 was considered statistically significant (in bold). Benjamini-Hochberg adjustment of multiple comparisons confirmed the results of the *t*-test (significant values in bold).*

### Correlation Analyses

**FIGURE 6:**
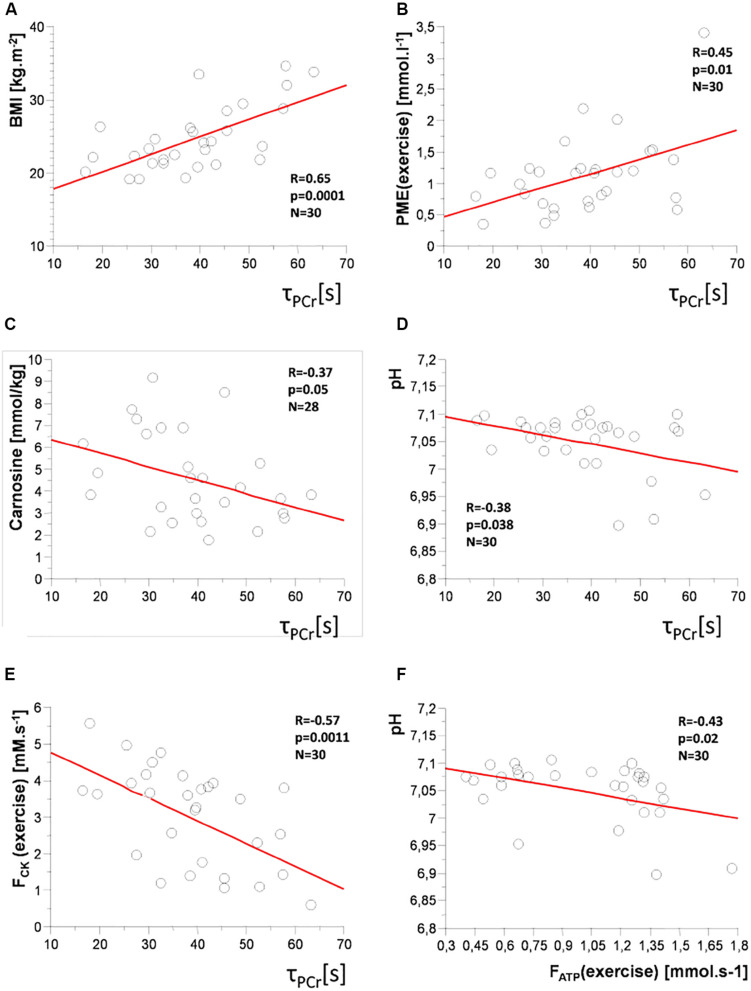
Correlation plots: time constant of PCr recovery (τ_*PCr*_) with **(A)** body mass index (BMI); **(B)** concentration of phosphomonoesters (PME) during exercise; **(C)** muscle carnosine concentration; **(D)** pH; **(E)** PCr-to-ATP flux (F_*CK*_) during exercise, and **(F)** between Pi-to-ATP flux (F_*ATP*_) and pH during exercise.

A positive correlation of the time constant of PCr recovery (τ_*PCr*_) was found with age (*R* = 0.56; *p* = 0.001), BMI (*R* = 0.65; *p* = 0.0001; Figure 6A), body fat mass (*R* = 0.63; *p* = 0.0004), and PME concentration and F_*ATP*_ flux during steady-state submaximal exercise (*R* = 0.45; *p* = 0.01 and *R* = 0.35; *p* = 0.049), respectively, (Figure 6B). There were also negative correlations of τ_*PCr*_ with muscle carnosine content (*R* = −0.37; *p* = 0.047; Figure 6C), whole-body muscle mass (*R* = −0.55; *p* = 0.0023), pH (*R* = −0.38; *p* = 0.038; Figure 6D), F_*CK*_ flux during steady-state exercise (*R* = −0.57; *p* = 0.001; Figure 6E), and PDE/GPC ratio (*R* = −0.46; *p* = 0.01).

As the age is positively associated with BMI and body fat mass and negatively with muscle mass, we have corrected the correlations with these parameters for age. After this correction, the time constant of PCr recovery (τ_*PCr*_) positively correlated with BMI (*R* = 0.42; *p* = 0.011), and body fat mass (*R* = 0.46; *p* = 0.006) and negatively with whole-body muscle mass (*R* = −0.43; *p* = 0.013).

F_*ATP*_ flux during exercise positively correlated with age (*R* = 0.53; *p* = 0.001), V_*iPCr*_ (*R* = 0.62; *p* = 0.0002), Q_*max*_ (*R* = 0.43; *p* = 0.01), PME concentration during exercise (*R* = 0.54; *p* = 0.003), ΔPME (*R* = 0.54; *p* = 0.003), PDE concentration at rest (*R* = 0.43; *p* = 0.02) and during exercise (*R* = 0.40; *p* = 0.03), and GPC concentration (*R* = 0.51; *p* = 0.004) but negatively with pH (*R* = −0.43; *p* = 0.02; Figure 6F), and PDE/GPC ratio (*R* = −0.37; *p* = 0.04).

The ratio of Pi_2_/Pi which provides information about mitochondrial density associated positively with concentration of carnosine (*R* = 0.477; *p* = 0.01) and negatively with V_*iPCr*_ (*R* = −0.41; *p* = 0.02).

PME concentration during the exercise period positively correlated with age (*R* = 0.40; *p* = 0.03), initial recovery rate V_*iPCr*_ (*R* = 0.49; *p* = 0.007) and with F_*ATP*_ flux during exercise (*R* = 0.54; *p* = 0.003), and ΔF_*ATP*_ (*R* = 0.45; *p* = 0.01). On the other hand, there is a negative correlation with F_*CK*_ flux during exercise (*R* = −0.56; *p* = 0.001), PDE/GPC ratio (*R* = −0.41; *p* = 0.03), and PCr/Pi ratio (*R* = −0.40; *p* = 0.03).

**FIGURE 7:**
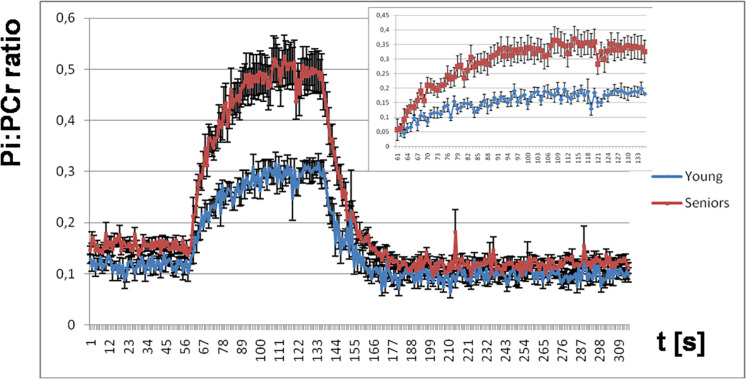
The time course of the Pi:PCr ratio during the period of rest, exercise, and recovery. Highlighted is the increase in the Pi:PCr ratio during exercise for both groups adjusted at basal conditions.

The Pi:PCr ratio attained during exercise was significantly lower in young people than in seniors, and we observed also a faster Pi:PCr kinetics in young subjects compared to seniors during the recovery period. Mean and SEM data for the time-course of the Pi:PCr ratio during the period of rest, exercise, and recovery in the group of young subjects and seniors are depicted in Figure 7. There is also a comparison of the increase in Pi:PCr ratio between both groups adjusted at basal conditions because we observed a lower Pi:PCr ratio in the young group at rest.

## Discussion

Our study focused on the detection of possible differences in ATP production in skeletal muscles of young volunteers and seniors. We have also investigated the potential differences associated with varying contents of carnosine as an important compound allocated in skeletal muscle. We found a longer PCr recovery after exercise in the group of seniors, who had also a lower capacity to cope as indicated by a strong increase in PME concentration as well as lower muscle carnosine concentration and its negative correlation with age. Exercise led in both groups to an increase in Pi which was connected with a larger decrease in k_*ATP*_ in young subjects.

Aging is in general associated with lower physical activity and worse physical fitness. In our study, elderly subjects displayed longer PCr recovery after exercise (τ_*PCr*_) compared to young people, which is in agreement with lower training status and lower habitual physical activity and fitness in seniors reported in the literature (Ayabe et al., 2009; Hawkins et al., 2009; Speakman and Westerterp, 2010; Buchman et al., 2014; Boulton et al., 2018). Furthermore, τ_*PCr*_ correlated positively with age (*R* = 0.56, *p* = 0.001), as well as BMI (*R* = 0.65, *p* = 0.0001). Specifically in seniors, we observed a negative correlation in Q_*max*_ with BMI (*R* = −0.57; *p* = 0.01). This is in good agreement with the observation of lower Q_*max*_ in obese subjects (Valkovič et al., 2016). A positive association of τ_*PCr*_ with body fat mass (*R* = 0.56, *p* = 0.01) and a negative correlation with muscle mass (*R* = −0.55, *p* = 0.02) also agreed with the interrelation of τ_*PCr*_ with physical fitness.

Exercise challenge was in seniors linked to a strong increase in PME concentration (*p* = 0.006). In young subjects, we have observed only a tendency to increase in muscle PME levels, which did not reach significance (*p* = 0.62). This observation can be explained by better capacity of young muscle to cope with workload because an increase in PME may be attributed to an accumulation of IMP (Soussi et al., 1990), which resonates in the PME frequency range (Van Wazer and Ditchfield, 1987). Newcomer and Boska (Newcomer and Boska, 1997) showed that about 1–3-mmol/l increase in PME resonance may be partly attributed to an increase in IMP. Accumulation of IMP is connected with production of NH_3_ in the muscle and has been postulated to be related to metabolic stress (Sahlin, 1992). Thus, our observations are in agreement with better physical fitness and better ability to cope with exercise load of muscle in the group of young subjects than in seniors. This is also supported by the association of Pi-to-ATP flux with PME concentration during exercise (*R* = 0.54, *p* = 0.003), which agrees with Sleigh et al. (2016). The concentration of glucose-6-phosphate, resonating close to PME frequency (Rothman et al., 1992), might also be increased during exercise. In particular, due to the age-dependent decrease in glucose-6-phosphate dehydrogenase (G6PD) activity, glucose-6-phosphate could be metabolized slower resulting in higher oxidative stress in the old-age population (Nikolaidis et al., 2013; Maurya et al., 2016).

Aging is also associated with a loss of skeletal muscle mass, which was shown in biopsy studies to be connected also with significant reduction in skeletal muscle carnosine content (Stuerenburg and Kunze, 1999; Tallon et al., 2007). Carnosine is in humans dominantly allocated in muscle tissue with positive effects on muscle strength and pH buffering properties. In accordance with these literature findings, we observed a significantly lower muscle concentration of carnosine in seniors (*p* = 0.0004) using non-invasive ^1^H-MRS. Furthermore, we have found a negative association of muscle carnosine concentration with age (*R* = 0.57, *p* = 0.001). Loss of muscle mass is also associated with a loss of skeletal muscle function, i.e., a decrease in its capacity to undertake anaerobic activity. We documented a positive correlation of carnosine with Pi_2_ (*R* = 0.60, *p* = 0.007) and also with the Pi_2_/Pi ratio (*R* = 0.513, *p* = 0.02) in seniors and with the Pi_2_/Pi ratio (*R* = 0.48, *p* = 0.01) in the whole study population. These findings together with lower muscle carnosine concentration in seniors are also in agreement with the reported lower Pi_2_/Pi ratio in the overweight-to-obese sedentary subjects than in lean active individuals (Valkovič et al., 2016).

It was also reported that a major determinant of carnosine levels is muscle fiber type. Fast-twitch fibers contain twice as much carnosine as the slow-twitch fibers (Dunnett et al., 1997; Hill et al., 2007). Low muscle carnosine content was found in marathon runners, and moderately positive correlations between fast-twitched fiber proportion and carnosine content were shown using muscle biopsies in untrained subjects (Mannion et al., 1995; Suzuki et al., 2002; Puri, 2006). Loss of fast-twitch muscle fibers is in rodents an early marker of muscle aging (Ishihara and Araki, 1988). The age-related degeneration of the glycolytic fibers, responsible for muscle contraction power, indicates that lower power in seniors could reflect proportions of glycolytic fiber loss and therefore lowering of the muscle carnosine content.

The state of intracellular oxidative phosphorylation could be derived also from the Pi:PCr ratio, which is also an indicator of ADP in the myocyte (Chance et al., 1988). Even though we have normalized the exercise load for our volunteers to 30% of maximal voluntary contraction, the Pi:PCr ratio attained during exercise was significantly lower in young people than in the elderly and we observed also faster Pi:PCr kinetics in young subjects compared to seniors during the recovery period. If we assume that ADP regulates a cellular respiration rate, the rapid decrease in the Pi:PCr ratio during the recovery period could have two possible explanations: (i) the rate of mitochondrial activity increased sufficiently or (ii) the muscle blood supply was more than sufficient to meet the muscle oxygen demand (Yoshida and Watari, 1992). Our observations are in agreement with better training status and higher capacity to cope with the same workload in young subjects.

Moreover, our observation of a significantly lower pH after exercise (*p* = 0.0183) in seniors and a negative association of pH with PCr recovery after exercise (τ_*PCr*_; *R* = −0.37; *p* = 0.0382) agrees with Iotti et al. (1993) and Layec et al. (2013) and points out to slower kinetics during the postexercise recovery period.

In both subject groups, there was a strong increase in Pi concentration with exercise. This increase was connected with a decrease in k_*ATP*_, but only in young subjects similarly as suggested in a pilot examination by Tušek Jelenc et al. (2016). This could potentially again point toward lower metabolic ability of the muscle to match the exercise-driven metabolic demand in the elderly and/or a generally higher demand for ATP production in the muscle of the elderly volunteers. The requirement of higher ATP production may be related also to significantly higher Pi-to-ATP flux in seniors. Thus, the muscle of elderly individuals needs to respond to the similar exercise load with higher effort, which is represented with a higher Pi-to-ATP flux. The magnitude of the Pi-to-ATP flux in the group of young volunteers at rest as well as increment during exercise is in agreement with previous reports (Sleigh et al., 2016; Tušek Jelenc et al., 2016). In this subpopulation, we also detected a correlation between recovery-derived Q_*max*_ and F_*ATP*_ during exercise (*R* = 0.43; *p* = 0.011) and also ΔF_*ATP*_ (*R* = 0.34; *p* = 0.048).

The possibility to measure both parameters (Q_*max*_ and F_*ATP*_ during exercise) in a single exercise-recovery experiment is of particular importance when elderly subjects are scanned as, due to their lower physical fitness, a long recovery period between several exercise bouts would be required.

This study has certain limitations, which included a relatively low number of volunteers in both study groups. Another limitation is that the senior group consisted of volunteers with various physical fitness and training states, but this is true also for the young participants and thus should not influence the intergroup comparisons.

We can summarize that both excessive Pi-to-ATP flux and an increase in PME concentration during exercise in seniors refer to the lower ability to cope with the exercise load compared to young subjects. Moreover, the muscle of young volunteers had faster recovery kinetics during the postexercise recovery phase. These findings could be related also with a lower muscle carnosine concentration and lower pH after exercise in elderly volunteers. This work demonstrates a well-tolerated multi-parametric multinuclear MRS protocol for the assessment of muscle energy metabolism *in vivo*, paving a way for large population studies in the future.

## Data Availability Statement

The datasets generated for this study are available on request to the corresponding author.

## Ethics Statement

The studies involving human participants were reviewed and approved by Ethical commission of Medical University of Vienna (EK 754/2011). All participants provided their written informed consent to participate in this study.

## Author Contributions

JU, BU, MK, RK, and LV contributed to the conception and design of the study. PK, RK, IJ, and MTJ acquired the data. PK, MTJ, and LV performed data analysis. MK and LV interpreted the data. PK drafted the manuscript. RK, IF, JU, ST, MK, and LV revised the draft critically for important intellectual content. All authors contributed to the manuscript revision and read and approved the final version.

## Conflict of Interest

The authors declare that the research was conducted in the absence of any commercial or financial relationships that could be construed as a potential conflict of interest.
